# Role of *HIF1A*, *VEGFA* and *VEGFR2* SNPs in the Susceptibility and Progression of COPD in a Spanish Population

**DOI:** 10.1371/journal.pone.0154998

**Published:** 2016-05-10

**Authors:** Rebeca Baz-Dávila, Adriana Espinoza-Jiménez, María del Cristo Rodríguez-Pérez, Javier Zulueta, Nerea Varo, Ángela Montejo, Delia Almeida-González, Armando Aguirre-Jaime, Elizabeth Córdoba-Lanús, Ciro Casanova

**Affiliations:** 1 Research Unit, Hospital Universitario Nuestra Señora de Candelaria, Santa Cruz de Tenerife, Spain; 2 Pulmonary Department, Clínica Universitaria de Navarra, Pamplona, Spain; 3 Biochemical Analysis Department, Clínica Universitaria de Navarra, Pamplona, Spain; 4 Pulmonary Department, Hospital Universitario Nuestra Señora de Candelaria, Santa Cruz de Tenerife, Spain; 5 Immunology Department, Hospital Universitario Nuestra Señora de Candelaria, Santa Cruz de Tenerife, Spain; University of Birmingham, UNITED KINGDOM

## Abstract

Hypoxia is involved in the development of chronic inflammatory processes. Under hypoxic conditions HIF1A, VEGF and VEGFR2 are expressed and mediate the course of the resultant disease. The aim of the present study was to define the associations between tSNPs in these genes and COPD susceptibility and progression in a Spanish cohort. The T alleles in rs3025020 and rs833070 SNPs (*VEGFA* gene) were less frequent in the group of COPD cases and were associated with a lower risk of developing the disease (OR = 0.60; 95% CI = 0. 39–0.93; p = 0.023 and OR = 0.60; 95% CI = 0.38–0.96; p = 0.034, respectively) under a dominant model of inheritance. The haplotype in which both SNPs presented the T allele confirmed the association found (OR = 0.02; 95% CI = 0.00 to 0.66; p = 0.03). Moreover, patients with COPD carrying the T allele in homozygosis in rs3025020 SNP showed higher lung function values and this association remained constant during 3 years of follow-up. In conclusion, T allele in rs833070 and rs3025020 may confer a protective effect to COPD susceptibility in a Spanish population and the association of the SNP rs3025020 with lung function may be suggesting a role for VEGF in the progression of the disease.

## Introduction

Chronic Obstructive Pulmonary Disease (COPD) is a leading cause of death worldwide, mainly caused by tobacco smoking. As pulmonary function deteriorates, and as the disease progresses, the risk of alveolar hypoxia and consequent hypoxemia increase. The principal contributor to hypoxemia in COPD patients is ventilation/perfusion (V/Q) mismatch resulting from progressive airflow limitation and emphysematous destruction of the pulmonary capillary bed. It now seems clear that tissue hypoxia is a key player in many of the processes and extrapulmonary comorbidities that characterize COPD.

The chronic hypoxemia could promote the expression of proteins that are involved in the transport of oxygen (erythropoietin), in the vasculogenesis and angiogenesis (vascular endothelial growth factor, VEGF and its receptors) or by the stimulation of expression of other genes (hypoxia inducible factor -1, HIF-1). HIF-1 is a transcription factor that acts as principal regulator of oxygen homeostasis, playing a fundamental role in the physiological response to hypoxia. It is a heterodimer composed of two subunits: HIF-1α and HIF-1β. HIF-1β is a constitutive core protein, whereas expression of HIF-1α is regulated by oxygen concentration. Furthermore, there is evidence that hypoxia is involved in the development of chronic inflammatory processes [1] and factor hypoxia-1 inducible acts as a regulator of the development of inflammation [2,3]. In fact, HIF-1 can also be activated in response to several inflammatory stimuli [4,5]. Nevertheless, the possible involvement of genetic alterations in *HIF1A* has not been previously studied in the COPD inflammation process caused by tobacco. VEGF is one of the most important angiogenic factors. Its action depends on its interaction with specific receptors, mainly VEGFR2 (also known as KDR). These proteins are expressed in different cell types and organs, being the alveoli and pulmonary epithelium the areas with higher expression in patients with COPD [6]. Under hypoxic conditions, *VEGFA* and *VEGFR2* gene expression are regulated by the action of HIF-1, acting in its specific binding site in the promoter. There are three genetic association studies that attempted to explore the relationship between polymorphisms in the *HIF1A* and *VEGFA* genes and the risk of COPD in a Japanese [7] and Chinese [8,9] populations, with negative results. However, Ding *et al*., found an haplotype in *VEGFA* gene that could be associated with an increased risk of COPD in Chinese population [9]. Furthermore, Sharma *et al*. [10] had described an association of SNPs in *VEGFA* gene with FEV_1_/FVC when studied the progress of asthma in children and Simpson *et al*. [11] study in population-based and asthma cohorts suggest an important role of *VEGFA* SNPs in airway function at different ages.

The aim of the present study was to determine whether Single Nucleotide Polymorphisms (SNPs) in *HIF1A*, *VEGFA* and *VEGFR2* genes are associated with susceptibility and development of COPD in a Spanish population.

## Materials and Methods

### Study population and clinical parameters

Participants in the study were divided into three groups: patients with COPD, smokers without COPD and nonsmoking controls without respiratory disease (healthy controls).

Patients with COPD were recruited from the pulmonary clinic at the Hospital Universitario Nuestra Señora de Candelaria from Tenerife (Spain) since 1997. Inclusion criteria were: ≥35 years, smoking history of ≥20 pack-year and FEV_1_/FVC ratio <0.7 measured 20 minutes after administration of 400 mg of inhaled albuterol. In addition, patients were clinically stable for at least 6 weeks prior to the evaluation of respiratory symptoms, lung function and sample collection. Individuals were excluded if they had history of other diseases like asthma or bronchiectasis, and if they had physical or mental inability to perform different tests. The control group of current smokers without COPD were selected, mostly in the pulmonary clinic at HUNSC and a small proportion was obtained from an adult general population cohort (CDC), from the Canary Islands [12] created to study the 3 most prevalent diseases in the Canary Islands: cardiovascular disease, diabetes and cancer. The inclusion criteria for this control group were ≥35 years, smoking history of ≥ 15 pack-year and normal lung function, defined as post bronchodilator FEV_1_/FVC ratio ≥ 0.7 and absence of lung diseases. As a second control group, we included healthy individuals without smoking history composed entirely by patients ≥ 35 years from the CDC study cited above.

In order to minimize any possible effect of population structure on our estimations, cases and control individuals were recruited if they have at least two generations of Canarian ancestry [13].

This work had the approval of the Ethics Committee of Clinical Investigation of the Hospital Universitario Nuestra Señora de Candelaria (PI-10/08, PI-06/09) and the written inform consent of all patients.

In terms of the clinical parameters, patients with COPD were interviewed once a year. At each visit clinical variables were recorded as follows: nutritional assessment was performed using the body mass index (BMI) calculated as the ratio of weight in kg and height in meters squared. Pulmonary function tests were performed following American Thoracic Society guidelines [14]. Diffusion capacity for carbon monoxide was determined with the single-breath technique following the European Respiratory Society/American Thoracic Society guidelines [15]. The arterial oxygen tension (PaO_2_) was measured at rest and dyspnea was assessed using the modified MRC scale (mMRC) [16]. Exercise capacity was also tested using the best of two 6-min walking distance (6MWD) tests separated by at least 30 min following the ATS recommendations [17]. The BODE (Body Mass Index, Airflow Obstruction, Dyspnea, Exercise Performance) Index was calculated as previously described [18].

### Genotyping and serum measurements

TagSNPs in the candidate genes were identified using the Haploview program (v4.1) [19] and data from Caucasian population available in SeattleSNP (http://pga.gs.whashington.edu/) and *HapMap* (http://hapmap.ncbi.nlm.nih.gov/) databases. A multimarker tagging algorithm with criteria of r^2^ cutoff for linkage disequilibrium clustering set to 0.8 and minor allele frequency (MAF) ≥ 0.1 were used. Additionally, other polymorphisms were included in the study due to its position in the gene or the relevant information provided in the scientific literature.

DNA was extracted from peripheral blood samples using commercial Illustra Blood Genomic Prep Mini Spin Kit (GE Healthcare) according to the manufacturer's instructions. Genotyping was performed using iPlex-Gold (Sequenom) platform at the National Genotyping Center (CeGen-ISCIII). Cases and controls were randomly placed on the plates, so that it was a double-blind trial. Arrays with the results of the analysis were downloaded from the SNPator platform [20]. A random 10% of samples were sequenced to confirm the results of genotyping using an *ABI Prism 310* Genetic Analyzer (Applied Biosystems).

Serum of COPD patients was separated from whole blood by centrifugation (3200 rpm) and aliquots were stored at -80°C until laboratory analysis. TNF-α and VEGF levels in serum were determined by noncompetitive enzyme-linked immunosorbent assay (ELISA), using different available commercial kits (R&D Systems Inc.) following the manufacturer's instructions. The within-assay coefficient of variation for all assays was less than 10%.

### Replication study and statistical analysis

197 COPD patients and 192 smokers without COPD were kindly donated from Clínica Universitaria de Navarra, and 500 nonsmoking controls from National DNA Bank, and used for genotyping by the iPlex-Gold platform. These individuals were selected following the same inclusion and exclusion criteria previously described.

Statistical analysis was performed using SPSS (v.21) (IBM SPSS Statistics for Windows, Armonk- IBM Corp.). ANOVA, Student *t*, Kruskal-Wallis, Mann-Whitney U and χ2 Pearson tests were used as appropriate. Multivariate logistic regression analysis was used to adjust for independent predictors (age, gender, pack-years of smoking) in each case. General linear model (GLM) for repeated measures was used to assess disease progression from longitudinal clinical and lung data. For genetic analysis, χ2 Pearson test was used for comparisons of allelic and genotypic distribution between patients with COPD and the two control groups. The values of linkage disequilibrium (LD) and Hardy-Weinberg were calculated using GENEPOP v3.4 [21, 22] and Haploview (v4.1) [18]. The haplotype analysis was performed using PHASE program (v2.1) [23]. To determine the associations and verify the consistency of the inferred data, haplotypes were reconstructed from the best average goodness-of-fit output of six runs with 1,000 permutations. A multivariate logistic regression analysis was performed to estimate the odds ratio (OR) specific for each genotype, with a confidence interval (CI) of 95%, in susceptibility to COPD, using the SNPStats software [24]. In order to reduce potential bias generated by the use of multiple tests, we estimate the effective number of independent test in every case where significant differences existed between patients and controls [25]. In all tests, bilateral contrast hypothesis was considered significant p-value less than 0.05.

## Results

The demographic and baseline characteristics of the groups are summarized in **Table 1**. 725 samples were successfully genotyped: 189 patients with COPD and 536 control subjects. COPD patients (139 men and 50 women) were mainly in mild to moderate stage of the disease (56% in GOLD stages I- II).

**Table 1:** Demographic characteristics of individuals included in the association study at baseline.

Variable	COPD patients (n = 142)	Smoking controls (n = 77)	Nonsmoking controls (n = 459)	p-value
**Gender (% male)**	74	50	63	<0.0001
Age (years)*	63 ± 10	48 ± 10	50 ± 9	<0.0001
**Smoking history (pack-yrs)**^†^*	64 ± 28	35 ± 14	0	<0.0001
**FEV**_**1**_ (L)*	1.50 ± 0.7	3 ± 0.7	__	NA
**FEV**_**1**_ (% pred)*	56 ± 20	101 ± 14	__	NA
FVC (%)*	86 ± 22	107 ± 15	__	NA
**FEV**_**1**_/FVC*	51 ± 12	78 ± 7	__	NA
**PaO**_**2**_ (mmHg)*	69.3 ± 13.5	__	__	NA
**IC/TLC (%)**	34 ± 12	__	__	NA
**K**_**CO**_	58 ± 7	__	__	NA
6MWD (m)*	489 ± 89	__	__	NA
**BMI (Kg/m**^**2**^**)** *	28 ± 6	__	__	NA
mMRC dysnea**	1 (0–2)	__	__	NA
BODE index**	2 (0–3)	__	__	NA
**BODE index ≤ 2**^**‡**^	103	__	__	NA
**BODE index > 2**^**‡**^	46	__	__	NA
**GOLD index I-II**^**‡**^	106	__	__	NA
**GOLD index III-IV**^**‡**^	83	__	__	NA

* Data are presented as mean ± SD.

** Data are presented as median (P_25-75_).

^†^ Number of packs of cigarettes smoked per day x number of years smoking.

^**‡**^ Number of subjects in the two groups of GOLD and BODE index considered to analysis.

Median was used as cutoff in the BODE index. FEV_1_: forced expiratory volume in one second; FVC: forced expiratory volume; % pred: predicted percentage; 6MWD: six-min walk distance test; BMI: body mass index; IC: inspiratory capacity, TLC: total lung capacity; PaO_2:_ partial pressure of oxygen in arterial blood: NA: Not Applicable.

21 tSNPs (7 in *HIF1A*, 11 in *VEGFA* and 3 in *VEGFR2* genes) were analyzed. All SNPs were in Hardy-Weinberg equilibrium except rs2071559 in *VEGFR2* gene, which was then excluded from further analysis.

The SNPs studied in *HIF1A* and *VEGFR2* genes showed no significant differences in their frequency distribution within individuals with COPD when compared to both control groups **(Tables 2 and 3)**.

**Table 2:** Association of *HIF1A* polymorphisms with COPD.

SNP	Minor Allele	COPD patients MAF (%)	Nonsmoking MAF (%)	Smoking controls MAF (%)	OR_aj_ (95% CI)^a^	p-value	OR_aj_ (95% CI)^a^	p-value
rs2301106	C	0.14	0.14	0.10	1.23 (0.80–1.89)	>0.05	1.86 (0.84–4.12)	>0.05
rs12434438	G	0.33	0.32	0.27	1.16 (0.84–1.59)	>0.05	1.29 (0.76–2.16)	>0.05
rs11158358	G	0.21	0.21	0.18	1.20 (0.82–1.76)	>0.05	1.30 (0.70–2.41)	>0.05
rs10873142	C	0.32	0.28	0.24	1.27 (0.91–1.77)	>0.05	1.44 (0.83–2.49)	>0.05
rs41508050	T	0.01	0.01	0.00	0.66 (0.13–3.39)	>0.05	NA (0.00-NA)*	>0.05
rs2301113	C	0.37	0.33	0.29	1.20 (0.87–1.64)	>0.05	1.29 (0.74–2.33)	>0.05
rs4902080	T	0.07	0.08	0.09	0.93 (0.52–1.66)	>0.05	0.65 (0.27–1.55)	>0.05

^a^ COPD patients vs. nonsmokers.

^b^ COPD patients vs. smokers.

Data are presented as MAF: minor allele frequency; %: percentage; OR_ad_: adjusted odds ratio; CI: confidence interval. Age, gender and pack-year were included in a multivariate logistic regression analyses as potential independent predictors in an additive model.

* There is not enough individuals with TT genotype to complete the model.

**Table 3:** Association of *VEGFA* and *VEGFR2* polymorphisms with COPD.

SNP	Minor Allele	COPD patients MAF (%)	Nonsmoking controls MAF (%)	Smoking controls MAF (%)	OR_aj_ (95% CI)^a^	p-value	OR_aj_ (95% CI) ^b^	p-value
rs833069	C	0.41	0.38	0.41	1.09 (0.81–1.47)	>0.05	1.04 (0.60–1.80)	>0.05
rs833070	T	0.43	0.47	0.41	0.60 (0.38–0.96)	0.034*	1.15 (0.67–1.95)	>0.05
rs3025007	T	0.40	0.39	0.39	0.96 (0.70–1.30)	>0.05	0.85 (0.50–1.44)	>0.05
rs3025009	G	0.43	0.42	0.43	1.07 (0.79–1.44)	>0.05	1.00 (0.58–1.71)	>0.05
rs3025010	C	0.33	0.37	0.37	0.84 (0.60–1.16)	>0.05	1.02 (0.58–1.79)	>0.05
rs3025012	G	0.14	0.13	0.13	1.27 (0.83–1.93)	>0.05	1.23 (0.60–2.51)	>0.05
rs3025020	T	0.25	0.31	0.22	0.60 (0.39–0.93)	0.023*	1.19 (0.61–2.33)	>0.05
rs3025032	T	0.32	0.32	0.35	1.19 (0.86–1.64)	>0.05	1.45 (0.80–2.62)	>0.05
rs3025033	G	0.18	0.15	0.17	1.16 (0.78–1.75)	>0.05	0.95 (0.49–1.83)	>0.05
rs3025039	T	0.14	0.13	0.16	1.08 (0.69–1.69)	>0.05	0.87 (0.41–1.87)	>0.05
rs10434	A	0.42	0.43	0.43	1.09 (0.80–1.48)	>0.05	1.35 (0.81–2.27)	>0.05
rs1870377	A	0.20	0.19	0.24	1.17 (0.79–1.73)	>0.05	0.96 (0.51–1.81)	>0.05
rs2305948	T	0.12	0.12	0.11	1.08 (0.68–1.73)	>0.05	0.95 (0.41–2.16)	>0.05

^a^ COPD patients vs. nonsmokers.

^b^ COPD patients vs. smokers.

Data are presented as MAF: minor allele frequency; %: percentage; OR_ad_: adjusted odds ratio; CI: confidence interval. Age, gender and pack-year were included in a multivariate logistic regression analyses as potential independent predictors in an additive model.

*p<0.05 under a dominant model of inheritance.

In the *VEGFA* gene, we found that the T allele in rs3025020 and rs833070 polymorphisms was more frequent in the nonsmoking control group when comparing to the group of patients with COPD. The presence of this allele in both SNPs was associated with a lower risk of developing the disease (OR = 0.60; 95% CI = 0. 39–0.93; p = 0.023 for rs3025020 and OR = 0.60; 95% CI = 0.38–0.96; p = 0.034, for rs833070) under a dominant model of inheritance, and after correcting for independent predictors (gender, age and pack-year) in a multivariate analysis **(Table 3)**. These results were not observed when comparing the group of patients with COPD and the one of smokers without the disease. After correcting for multiple testing by using the effective number of independent test (9.72), the association of rs3025020 and rs833070 with susceptibility to COPD lost significance (adjusted values: p >0.05).

No differences in the frequency distribution of haplotypes in *HIF1A* and *VEGFR2* genes between cases and non-smoking controls (p>0.05) were observed **(S1 and S2 Tables)**. However, when comparing the inferred *VEGFA* gene haplotypes in the group of cases versus smoker controls, we found that the haplotype TTTATATCACG that appeared in our population with a frequency of 1.24%, was associated with a diminished COPD susceptibility (OR = 0.02; 95% CI = 0.00–0.66; p = 0.03) after correcting for age, gender and pack-year **(Table 4)**. This haplotype presented the less frequent alleles (T) of the SNPs rs833070 and rs3025020, which were firstly associated with COPD in the independent SNPs analysis. A list of inferred haplotypes of *VEGFA* gene are presented in the Supporting information section **(S3 Table)**.

**Table 4:** *VEGFA* haplotype association with COPD susceptibility.

	COPD patients vs. nonsmoking controls			COPD patients vs. smoking controls		
Haplotype^a^	Frequency (%)	OR_aj_ (95%CI)	p-value	Frequency (%)	OR_aj_ (95%CI)	p-value
CCCGTACTACA	13.78	1	__	16.27	1	__
TTTATATCACG	0.80	NA (NA-NA)	>0.05	1.24	0.02 (0.00–0.66)	0.03*

^a^ rs833069/ rs833070/ rs3025007/ rs3025009/ rs3025010/ rs3025012/ rs3025020/ rs3025032/ rs3025033/ r3025039/ rs10434.

Data are presented as: %: percentage; OR_aj_: adjusted odds ratio; CI; confidence interval; NA: not analyzed (due to one allele has a frequency = 0.00). Age, gender and pack-year were included in a multivariate logistic regression analyses as potential independent predictors in an additive model.

* p-value<0.05.

**Fig 1:**
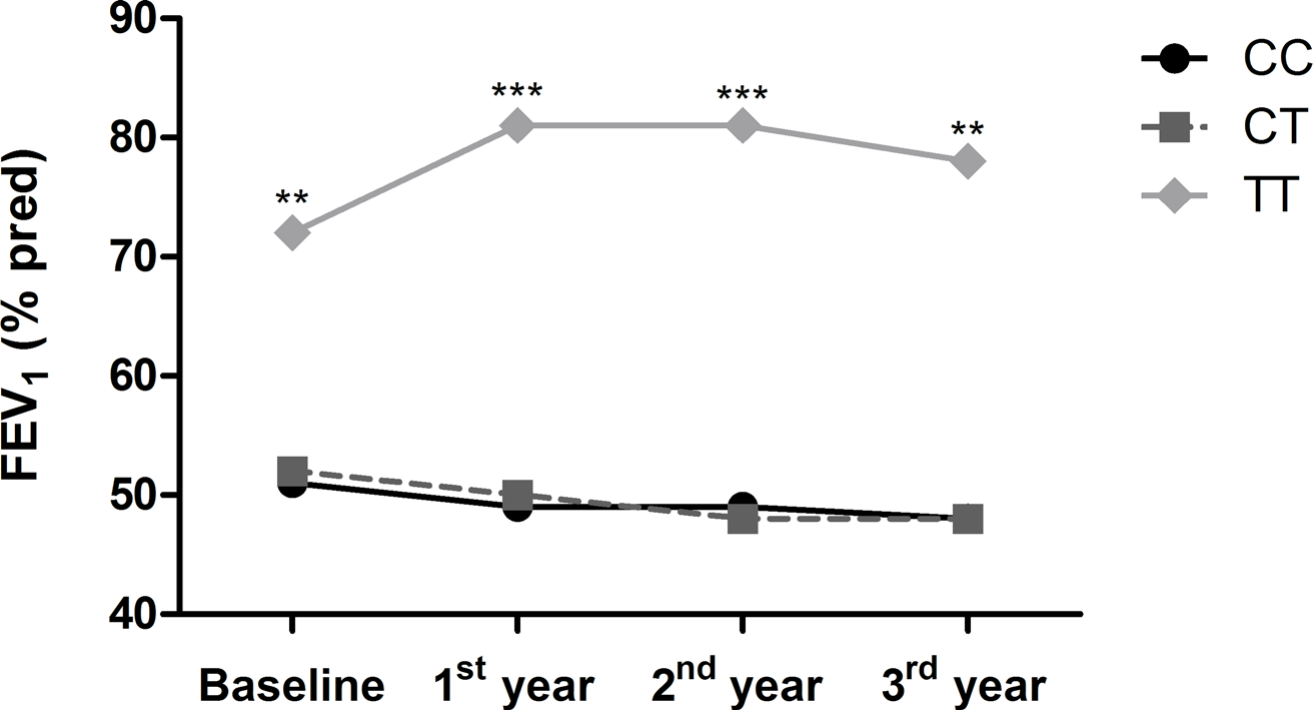
Lung function differences between rs3025020 genotypes in patients with Chronic Obstructive Pulmonary Disease over time. FEV_1_ (% pred) for the three genotypes of SNP rs3025020 (*VEGFA* gene) over time. The presence of allele T in homozygosis determined higher values of FEV_1_ (% pred) respect to the other genotypes. The differences remained constant for every visit (** p-value<0.01; *** p-value<0.001) and along 3 years of follow-up (p = 0.017). The sample size for each moment was: Baseline: N = 132; 1^st^ year: N = 118; 2^nd^ year: N = 98 and 3^rd^ year: N = 87.

Furthermore, COPD patients that carried the TT genotype of SNP rs3025020 presented higher lung function values overtime. We found that the presence of the minor allele T in homozygosis was significantly associated with a better lung function measured by FEV_1_ (% pred). This association remained constant over time when performing a longitudinal analysis of FEV_1_ during the 3-year follow-up period (p = 0.017) **(Fig 1)**.

By studying hypoxia responsive genes, we decided to subdivide the sample of patients with COPD according to their degree of hypoxemia (PaO_2_ <60mmHg, severe hypoxemia). We did not find any significant association between the analyzed SNPs and lung function variables within the groups of moderate and severe hypoxemia (p> 0.05).

None of the *HIF1A*, *VEGFA* or *VEGFR2* gene variants or haplotypes correlated with the serum inflammatory markers levels (p > 0.05).

Regarding to the replication study in another Spanish cohort, of the studied SNPs and haplotypes **(S4 and S5 Tables),** their distribution did not differ between the cases and controls groups (p> 0.05).

## Discussion

Our study explores the relationship between SNPs in the genes *HIF1A*, *VEGFA* and *VEGFR2* with COPD. Our major finding is that we found not only an association of an haplotype containing SNPs rs3025020 and rs833070 in the *VEGFA* gene with the susceptibility to disease, but also an association of SNP rs3025020 with the progression of COPD.

Specifically, the T alleles in both polymorphisms in the *VEGFA* gene were present with a higher frequency in the nonsmoking control group when compared to the patients with the disease group. This finding was supported by the fact that the haplotype, in which both SNPs present the T allele, was associated to a diminished COPD susceptibility. This is important because haplotypes are capable of detecting differences when individual SNPs lost significance due to small sample size. Furthermore, the presence of the TT genotype in rs3025020 seemed to be associated with a better lung function, given by higher levels of FEV_1_ (% pred) when compared to patients carrying the CT and CC genotypes. Interestingly, these differences were observed along the longitudinal 3-years follow up period. Previously, different SNPs were associated with lung function in population-based and asthma cohorts along time, suggesting a role of *VEGFA* variants in lung function [10, 11] and one of them, rs3025028, was also associated with the ratio of the active and inhibitory isoforms of VEGF-A_165_. Moreover, Ding *et al*. [9], described an haplotype of *VEGFA* gene potentially associated with an increased COPD risk in an Asian population. However, to our knowledge this is the first study reporting the longitudinal relationship between SNPs in *VEGFA* gene and disease progression in a well-characterized cohort of patients with COPD.

Several studies deepen the association of rs3025020 in *VEGFA* gene with VEGF secretion, with conflicting results. Ruggiero *et al*.,observed that TT genotype could increase serum levels of VEGF in three Caucasian populations [26]. However, this study shows heterogeneity of results depending on the population studied: polymorphisms associated with serum VEGF levels in a population, not replicated this association in other populations analyzed. Al-Habboubi *et al*. [27] find and association of T allele with higher serum levels of the protein in a healthy cohort. However, they could not find this association in a posterior study of vasoocclusive crisis in sickle cell disease [28]. According these results, we did not find association between VEGF serum levels and the studied SNPs within the group of patients with the disease. Furthermore, Boeck *et al*. (2015) [29] found that serum levels of VEGF measure at baseline, at stable and exacerbated patients with COPD, were not associated with clinically significant outcomes, but the VEGF course seems related to COPD prognosis. They observed that increased levels in VEGF over time were associated with the exacerbation frequency, the 1- and 2-year hospitalization rate as well as survival. Interestingly, Almawy *et al*. [30], suggest that the regulation of VEGF serum levels is not only dependent of genetic factors, but also depend on local and systemic factors accompanying the disease, which could explain our results.

Regarding the other studied SNPs, *VEGFA* polymorphism rs3025039 was previously studied in relation to COPD in a Japanese population, but without positive results [7]. According to this report, in our study we also analyzed the rs3025033 polymorphism, which is in linkage disequilibrium with rs3025039, and found no significant associations with disease susceptibility. Ding *et al*., also studied several SNPs of *VEGFA* gene, three of them included in our study (rs833070, rs3025033 and rs10434) in Chinese population, with negative results [9]. Interestingly, the difference between our results for SNP rs833070 and theirs could be due to differences in the genetic background of the studied populations.

To our knowledge, this is the first study that evaluated the association between *VEGFR2* gene variants and COPD, without any significant result. Similarly, Ding *et al*. carried on a study of *HIF1A* in Chinese population, but they also did not find significant differences for the studied SNPs between patients and control groups [8]. Moreover, we did not find any relationship between any variant of *HIF1A* and *VEGFR2* genes and clinical or lung function parameters in our COPD cohort in a cross sectional analysis.

This study has some limitations: firstly, a large sample size may be necessary principally in the case of smokers without the disease to discard a type II error. Secondly, VEGF serum levels were not measure in a control group to compare with the COPD patients group. Moreover, we cannot rule out that the VEGF serum levels of the patients observed may be influenced by the received treatment given to patients (such as inhaled corticosteroids) or by comorbidities. Finally, in our cohort, in order to reduce the possibility of population stratification individuals having at least two generations of canary ancestors were included. Along this line, we could not found significant association between the studied gene variants and susceptibility to disease in another cohort from Spain. The Canarian population has a particular genetic background, with African influence [31] which may hamper the replication of the results in a similar cohort. Nevertheless, this do not invalidate our previous findings.

In summary, this is the first study to assess the influence of *HIF1A* and *VEGFA* gene variants in the susceptibility and progression of COPD in a Spanish population. Based on a well-characterized cohort of patients with longitudinal follow-up the SNPs rs3025020 and rs833070 in the *VEGFA* gene were found associated with a lower risk susceptibility to the disease. In addition, the patients with COPD who presented the rs3025020 TT genotype had a better preservation of lung function for a long monitoring period suggesting a role for VEGF in the progression of COPD.

## Supporting Information

S1 Table Association study of *HIF1A* haplotypes and COPD. ^a^ rs2301106/ rs12434438/ rs11158358/ rs10873142/ rs41508050/ rs2301113/ rs4902080. Data are presented as MAF: minor allele frequency; %: percentage; OR_ad_: adjusted odds ratio; CI: confidence interval. Age, gender and pack-year were included in a multivariate logistic regression analyses as potential independent predictors in an additive model.
(PDF)

S2 Table Association study of *VEGFR2* haplotypes and COPD. ^a^ rs1870377/ rs2305948. Data are presented as MAF: minor allele frequency; %: percentage; OR_ad_: adjusted odds ratio; CI: confidence interval. Age, gender and pack-year were included in a multivariate logistic regression analyses as potential independent predictors in an additive model.
(PDF)

S3 Table Association study of *VEGFA* haplotypes and COPD. ^a^ rs833069/ rs833070/ rs3025007/ rs3025009/ rs3025010/ rs3025012/ rs3025020/ rs3025032/ rs3025033/ rs3025039/ rs10434. Data are presented as MAF: minor allele frequency; %: percentage; OR_ad_: adjusted odds ratio; CI: confidence interval. Age, gender and pack-year were included in a multivariate logistic regression analyses as potential independent predictors in an additive model.
(PDF)

S4 Table Replication study of associated SNPs in the *HIF1A*, *VEGFA* and *VEGFR2* genes with COPD. ^a^ COPD patients vs. Nonsmoking control group; ^b^ COPD patients vs. Smoking control group. Data are presented as MAF: minor allele frequency; %: percentage; OR_ad_: adjusted odds ratio; CI: confidence interval. Age, gender and pack-year were included in a multivariate logistic regression analyses as potential independent predictors in an additive model. * Nonsmoking control is not in Hardy-Weinberg equilibrium.
(PDF)

S5 Table Replication study of AATATATCACG haplotype in the *VEGFA* gene with COPD susceptibility. Data are presented as MAF: minor allele frequency; %: percentage; OR_ad_: adjusted odds ratio; CI: confidence interval; NA: not analyzed. Age, gender and pack-year were included in a multivariate logistic regression analyses as potential independent predictors in an additive model. ^a^ rs833069/ rs833070/ rs3025007/ rs3025009/ rs3025010/ rs3025012/ rs3025020/ rs3025032/ rs3025033/ rs3025039/ rs10434.
(PDF)

## References

[1] Taylor CT. Interdependent roles for hypoxia inducible factor and nuclear factor-kappaB in hypoxic inflammation. J Physiol 2008; 586: 4055–4059. 10.1113/jphysiol.2008.157669 18599532 PMC2652186

[2] Hellwig-Burgel T, Rutkowski K, Metzen E, Fandrey J, Jelkmann W. Interleukin-1beta and tumor necrosis factor-alpha stimulate DNA binding of hypoxia-inducible factor-1. Blood 1999; 94: 1561–1567. 10477681

[3] Scharte M, Han X, Bertges DJ, Fink MP and Delude RL. Cytokines induce HIF-1 DNA binding and the expression of HIF-1-dependent genes in cultured rat enterocytes. Am J Physiol Gastrointest Liver Physiol 2003; 284: 373–384. 10.1152/ajpgi.00076.2002 12388200

[4] Li QF and Dai AG. Hypoxia inducible factor-1 alpha correlates the expression of heme oxygenase 1 gene in pulmonary arteries of rat with hypoxia induced pulmonary hypertension. Acta Biochim Biophys Sin 2004; 36: 133–140. 14970910 10.1093/abbs/36.2.133

[5] Semenza GL. Pulmonary vascular responses to chronic hypoxia mediated by hypoxia-inducible Factor 1. Proc Am Thorac Soc 2005; 2 (1): 68–70. 16113471 10.1513/pats.200404-029MS

[6] Papaioannou A, Kostikas K, Kollia P, Gourgoulianis KI. Clinical implications for vascular endothelial growth factor in the lung: friend or foe? Respir Res 2006; 7:128 17044926 10.1186/1465-9921-7-128 PMC1629021

[7] Sakao S, Tatsumi K, Hashimoto T, Igari H, Shino Y, Shirasawa H et al Vascular endothelial growth factor and the risk of smoking-related COPD. Chest 2003; 124 (1): 323–327. 12853540 10.1378/chest.124.1.323

[8] Ding Y, Yang D, Xun X, Wang Z, Sun P, Xu D, et al Association of genetic polymorphisms with chronic obstructive pulmonary disease in the Hainan population: a case-control study. Int J COPD 2015; 10: 7–13. 10.2147/COPD.S73042 PMC4279605 25565795

[9] Ding Y, Yang D, Zhou L, Xu J, Chen Y, He P, et al Variants in multiple genes polymorphism association analysis of COPD in the Chinese Li population. Int J COPD 2015; 10: 1455–1463. 10.2147/COPD.S86721 PMC4524388 26251585

[10] Sharma S, Murphy AJ, Soto-Quiros ME, Avila L, Klanderman BJ, Sylvia JS, et al Association of VEGF polymorphisms with childhood asthma, lung function and airway responsiveness. Eur Respir J 2009; 33: 1287–1294. 10.1183/09031936.00113008 19196819 PMC3725278

[11] Simpson A, Custovic A, Tepper R, Graves P, Stern DA, Jones M, et al Genetic Variation in Vascular Endothelial Growth Factor-A and Lung Function. Am J Respir Crit Care Med 2012; 185 (11): 1197–1204. 10.1164/rccm.201112-2191OC 22461367 PMC3373065

[12] Cabrera de León A, Rodríguez-Pérez MC, Almeida-González D, Domínguez-Coello S, Aguirre-Jaime A, Brito-Díaz B, et al Presentación de la cohorte "CDC de Canarias": objetivos, diseño y resultados preliminares. Rev Esp Salud Pública 2008; 82 (5): 519–534. 19039505 10.1590/s1135-57272008000500007

[13] Maca-Meyer N, Villar J, Pérez-Méndez L, Cabrera de León A, Flores C. A tale of aborigines, conquerors and slaves: Alu insertion polymorphism ante peopling of Canary Islands. Ann Hum Genet 2004; 68 (Pt 6): 600–605. 15598218 10.1046/j.1529-8817.2003.00125.x

[14] American Thoracic Society Statement. Lung function testing: selection of reference values and interpretative strategies. Am Rev Respir Dis 1991; 144: 1202–1218. 1952453 10.1164/ajrccm/144.5.1202

[15] Macintyre N, Crapo RO, Viegi G, Johnson DX, van der Grinten CP, Brusasco V, et al Standardization of the single-breath determination of carbon monoxide uptake in the lung. Eur Respir J 2005; 26 (4): 720–35. 16204605 10.1183/09031936.05.00034905

[16] Mahler D, Wells C. Evaluation of clinical methods for rating dyspnea. Chest 1988; 93: 580e6. 3342669 10.1378/chest.93.3.580

[17] American Thoracic Society Statement. Guidelines for the Six-Minute Walk Test. Am J Respir Crit Care Med 2002; 166:111–117. 12091180 10.1164/ajrccm.166.1.at1102

[18] Celli BR, Cote CG, Marín JM, Casanova C, Montes de Oca M, Méndez RA, et al The body-mass index, airflow obstruction, dyspnea, and exercise capacity index in chronic obstructive pulmonary disease. N Engl J Med 2004; 350 (10): 1005–1012. 14999112 10.1056/NEJMoa021322

[19] Barret JC, Fry B, Maller J, Daly MJ. Haploview: analysis and visualization of LD and haplotype maps. Bioinformatics 2005; 21: 263–5. 15297300 10.1093/bioinformatics/bth457

[20] Morcillo- Suárez C, Alegre J, Sangros R, Gazave E, de Cid R, Milne R, et al SNP analysis to results (SNPator): a web-based environment oriented to statistical genomics analyses upon SNP data. Bioinformatics 2008; 24 (23): 2790–2791. 18515823 10.1093/bioinformatics/btn241

[21] Raymond M, Rousset F. GENEPOP (v. 1.2): population genetics software for exact tests and ecumenecism. J Hered. 1995; 86: 248–9.

[22] Rousset F. Genepop'007: a complete reimplementation of the Genepop software for Windows and Linux. Mol. Ecol. Resources 2008; 8: 103–106. 10.1111/j.1471-8286.2007.01931.x 21585727

[23] Stephens M, Donnelly P. A comparison of Bayesian methods for haplotype reconstruction. Am J Hum Genet 2003; 73: 1162–9. 14574645 10.1086/379378 PMC1180495

[24] Sole X, Guino E, Valls J, Iniesta R, Moreno V. SNPStats: a web tool for the analysis of association studies. Bioinformatics 2006; 22: 1928–9. 16720584 10.1093/bioinformatics/btl268

[25] Nyholt DR. A simple correction for multiple testing for SNPs in linkage disequilibrium with each other. Am J Hum Genet 2004; 74 (4): 765–769. 14997420 10.1086/383251 PMC1181954

[26] Ruggiero D, Dalmasso C, Nutile T, Sorice R, Dionisi L, Aversano M, et al Genetics of VEGF serum variation in human isolated populations of Cilento: importance of VEGF polymorphisms. PLoS One 2011; 6: e16982 10.1371/journal.pone.0016982 21347390 PMC3036731

[27] Al-Habboubi HH, Sater MS, Almawi AW, Al-Khateeb GM, Almawi WY. Contribution of VEGF polymorphisms to variation in VEGF serum levels in a healthy population. Eur Cytokine Netw 2011; (3):154–158. 10.1684/ecn.2011.0289 21982816

[28] Al-Habboubi HH, Mahdi N, Abu-Hijleh TM, Abu-Hijleh FM, Sater MS, Almawi WY. The relation of vascular endothelial growth factor (VEGF) gene polymorphisms on VEGF levels and the risk of vasoocclusive crisis in sickle cell disease. Eur J Haematol. 2012; 89 (5): 403–409. 10.1111/ejh.12003 22925497

[29] Boeck L, Mandal J, Costa L, Roth M, Tamm M, Stolz D. Longitudinal Measurement of Serum Vascular Endothelial Growth Factor in Patients with Chronic Obstructive Pulmonary Disease. Respiration 2015 [Epub ahead of print] 10.1159/000430993 26066063

[30] Almawi WY, Saldanha FL, Mahmood NA, Al-Zaman I, Sater MS, Mustafa FE. Relationship between VEGFA polymorphisms and serum VEGF protein levels and recurrent spontaneous miscarriage. Hum Reprod. 2013; 28 (10): 2628–2635. 10.1093/humrep/det308 23900206

[31] Fregel R, Gomes V, Gusmão L, González AM, Cabrera VM, Amorim A, et al Demographic history of Canary Islands male gene-pool: replacement of native lineages by European. BMC Evol Biol 2009; 9:181 10.1186/1471-2148-9-181 19650893 PMC2728732

