# Supplementary material for: Role of HIF1A, VEGFA and VEGFR2 SNPs in the Susceptibility and Progression of COPD in a Spanish Population
Source: PLoS One. 2016 May 10;11(5):e0154998. doi: 10.1371/journal.pone.0154998 (PMC4862690; doi:10.1371/journal.pone.0154998)
Supplement: S1 Table — a rs2301106/ rs12434438/ rs11158358/ rs10873142/ rs41508050/ rs2301113/ rs4902080. Data are presented as MAF: minor allele frequency; %: percentage; ORad: adjusted odds ratio; CI: confidence interval. Age, gender and pack-year were included in a multivariate logistic regression analyses as potential independent predictors in an additive model. (PDF) [file pone.0154998.s001.pdf]

S1 Table. Association study of *HIF1A* haplotypes and COPD.

Haplotype[a]	COPD patients vs. Nonsmoking controls			COPD patients vs. Smoking controls		
	Frequency (%)	OR_{aj} (95% CI)	p-value	Frequency (%)	OR_{aj} (95%CI)	p-value
TACTCAC	63.0	1	__	63.69	1	__
CGGCCCC	11.25	1.39 (0.87 - 2.22)	>0.05	11.24	2.26 (0.91 - 5.62)	>0.05
TGCCCCC	9.46	1.14 (0.69 - 1.90)	>0.05	10.32	1.03 (0.45 - 2.38)	>0.05
TGGCCCT	7.24	1.03 (0.55 - 1.94)	>0.05	6.65	0.84 (0.31 - 2.30)	>0.05
TACTCCC	4.39	0.96 (0.44 - 2.10)	>0.05	4.46	0.71 (0.21 - 2.42)	>0.05
CGGTCAC	2.34	0.57 (0.15 - 2.08)	>0.05	1.61	0.52 (0.08 - 3.24)	>0.05
TGCCTCC	1.16	0.84 (0.16 - 4.49)	>0.05	0.00	NA (NA - NA)	__
Others	1.16	0.88 (0.23 - 3.36)	>0.05	1.85	0.82 (0.16 - 4.26)	>0.05

[a] rs2301106/ rs12434438/ rs11158358/ rs10873142/ rs41508050/ rs2301113/ rs4902080. Data are presented as MAF: minor allele frequency; %: percentage; OR_{ad}: adjusted odds ratio; CI: confidence interval. Age, gender and pack-year were included in a multivariate logistic regression analyses as potential independent predictors in an additive model.
