# Supplementary material for: Role of HIF1A, VEGFA and VEGFR2 SNPs in the Susceptibility and Progression of COPD in a Spanish Population
Source: PLoS One. 2016 May 10;11(5):e0154998. doi: 10.1371/journal.pone.0154998 (PMC4862690; doi:10.1371/journal.pone.0154998)
Supplement: S2 Table — a rs1870377/ rs2305948. Data are presented as MAF: minor allele frequency; %: percentage; ORad: adjusted odds ratio; CI: confidence interval. Age, gender and pack-year were included in a multivariate logistic regression analyses as potential independent predictors in an additive model. (PDF) [file pone.0154998.s002.pdf]

S2 Table. Association study of *VEGFR2* haplotypes and COPD.

Haplotype[a]	COPD patients vs. Nonsmoking controls			COPD patients vs. Smoking controls		
	**Frequency (%)**	**OR_{aj} (95%CI)**	**p-value**	**Frequency (%)**	**OR_{aj} (95%CI)**	**p-value**
TC	71.29	1	__	69.26	1	__
AC	17.03	0.96 (0.60 - 1.56)	>0.05	18.18	1.35 (0.58 - 3.04)	>0.05
TT	9.18	0.84 (0.47 - 1.52)	>0.05	7.97	1.85 (0.46 - 7.52)	>0.05
AT	2.50	2. 80 (0.79 - 9.95)	>0.05	4.60	0.43 (0.08 - 2.40)	>0.05

[a] rs1870377/ rs2305948. Data are presented as MAF: minor allele frequency; %: percentage; OR_{ad}: adjusted odds ratio; CI: confidence interval. Age, gender and pack-year were included in a multivariate logistic regression analyses as potential independent predictors in an additive model.
