# Supplementary material for: Role of HIF1A, VEGFA and VEGFR2 SNPs in the Susceptibility and Progression of COPD in a Spanish Population
Source: PLoS One. 2016 May 10;11(5):e0154998. doi: 10.1371/journal.pone.0154998 (PMC4862690; doi:10.1371/journal.pone.0154998)
Supplement: S3 Table — a rs833069/ rs833070/ rs3025007/ rs3025009/ rs3025010/ rs3025012/ rs3025020/ rs3025032/ rs3025033/ rs3025039/ rs10434. Data are presented as MAF: minor allele frequency; %: percentage; ORad: adjusted odds ratio; CI: confidence interval. Age, gender and pack-year were included in a multivariate logistic regression analyses as potential independent predictors in an additive model. (PDF) [file pone.0154998.s003.pdf]

S3Table. Association study of *VEGFA* haplotypes and COPD.

Haplotype[a]	COPD patients vs. Nonsmoking controls			COPD patients vs. Smoking controls		
	Frequency (%)	OR_{aj} (95%CI)	p-value	Frequency (%)	OR_{aj} (95%CI)	p-value
CCCGTACTACA	13.84	1	__	16.42	1	__
CCCGTATCACG	10.75	0.68 (0.34 - 1.36)	>0.05	7.64	1.29 (0.19 - 8.96)	>0.05
TTTATACTACA	7.07	0.89 (0.42 - 1.86)	>0.05	4.57	1.18 (0.15 - 9.31)	>0.05
TTTACATCACG	6.97	0.49 (0.21 - 1.15)	>0.05	5.12	0.54 (0.10 - 2.85)	>0.05
CCCGTACCACG	5.17	1.66 (0.70 - 3.94)	>0.05	7.13	0.22 (0.04 - 1.16)	>0.05
CCCGTACCGTC	4.40	0.73 (0.26 - 2.04)	>0.05	3.22	0.13 (0.01 - 1.31)	>0.05
TTTACACCGTG	4.25	1.34 (0.51 - 3.53)	>0.05	5.68	1.97 (0.33 - 11.60)	>0.05
TTTATACCACA	3.68	1.02 (0.44 - 2.37)	>0.05	4.69	0.36 (0.06 - 2.02)	>0.05
TCTATACCACG	3.40	1.80 (0.65 - 4.96)	>0.05	6.30	0.36 (0.06 - 2.20)	>0.05
TCTATACTACA	3.14	2.03 (0.72 - 5.69)	>0.05	3.68	0.49 (0.09 - 2.61)	>0.05
TTCACGTCACG	3.11	1.70 (0.53 - 5.51)	>0.05	3.99	5.67 (0.41 - 78.03)	>0.05
TTCACGCTACA	2.86	1.26 (0.40 - 3.99)	>0.05	4.43	0.43 (0.08 - 2.41)	>0.05
TTCACGCCACG	2.81	0.44 (0.10 - 1.89)	>0.05	1.20	0.17 (0.01 - 4.97)	>0.05
TTCACACTACA	2.26	0.39 (0.09 - 1.69)	>0.05	2.04	0.86 (0.09 - 7.91)	>0.05
TCCGTACCACA	2.05	1.40 (0.41 - 4.70)	>0.05	2.33	0.49 (0.07 - 3.46)	>0.05
TTTACACCACG	1.88	0.37 (0.09 -1.58)	>0.05	1.80	0.08 (0.00 - 2.69)	>0.05
TTCACGCCGTG	1.82	1.65 (0.53 - 5.20)	>0.05	2.67	0.34 (0.04 - 2.77)	>0.05
TCTATATCACG	1.64	0.20 (0.03 - 1.51)	>0.05	1.81	0.07 (0.00 - 1.38)	>0.05
TTCACATCACG	1.46	0.26 (0.03 - 2.50)	>0.05	0.00	NA (NA - NA)	__
TCCGTATCACG	1.10	0.42 (0.07 - 2.54)	>0.05	0.00	NA (NA - NA)	__
TTCACATCACA	1.00	0.74 (0.13 - 4.34)	>0.05	0.00	NA (NA - NA)	__
CCCGTACCGCG	0.00	NA (NA - NA)	__	1.93	0.40 (0.04 - 4.44)	>0.05
TTTATATCACG	0.00	NA (NA - NA)	__	1.19	0.01 (0.00 - 0.57)	>0.05
TCCGTACCACG	0.00	NA (NA - NA)	__	1.13	0.38 (0.02 - 6.36)	>0.05
Others	15.32	0.64 (0.33 - 1.24)	>0.05	11.05	0.46 (0.10 - 2.07)	>0.05

[a] rs833069/ rs833070/ rs3025007/ rs3025009/ rs3025010/ rs3025012/ rs3025020/ rs3025032/ rs3025033/ rs3025039/ rs10434. Data are presented as MAF: minor allele frequency; %: percentage; OR_{ad}: adjusted odds ratio; CI: confidence interval. Age, gender and pack-year were included in a multivariate logistic regression analyses as potential independent predictors in an additive model.
