# Supplementary material for: Role of HIF1A, VEGFA and VEGFR2 SNPs in the Susceptibility and Progression of COPD in a Spanish Population
Source: PLoS One. 2016 May 10;11(5):e0154998. doi: 10.1371/journal.pone.0154998 (PMC4862690; doi:10.1371/journal.pone.0154998)
Supplement: S4 Table — a COPD patients vs. Nonsmoking control group; b COPD patients vs. Smoking control group. Data are presented as MAF: minor allele frequency; %: percentage; ORad: adjusted odds ratio; CI: confidence interval. Age, gender and pack-year were included in a multivariate logistic regression analyses as potential independent predictors in an additive model. * Nonsmoking control is not in Hardy-Weinberg equilibrium. (PDF) [file pone.0154998.s004.pdf]

S4 Table. Replication study of associated SNPs in the *HIF1A*, *VEGFA* and *VEGFR2* genes with COPD.

SNPs	Minor allele	COPD MAF (%)	Nonsmoking controls MAF (%)	Smoking controls MAF (%)	OR_{aj} (95%CI)[a]	p-value	OR_{aj} (95%CI)[b]	p-value
VEGFA								
rs833070	T	0.47	0.48	0.43	1.10 (0.75 - 1.62)	>0.05	1.18 (0.87 - 1.61)	>0.05
rs3025020	T	0.28	0.27	0.21	1.31 (0.83 - 2.05)	>0.05	1.14 (0.72 - 1.79)*	>0.05

[a] COPD patients vs. Nonsmoking control group; [b] COPD patients vs. Smoking control group. Data are presented as MAF: minor allele frequency; %: percentage; OR_{ad}: adjusted odds ratio; CI: confidence interval. Age, gender and pack-year were included in a multivariate logistic regression analyses as potential independent predictors in an additive model. * Nonsmoking control is not in Hardy-Weinberg equilibrium.
