# Supplementary material for: Role of HIF1A, VEGFA and VEGFR2 SNPs in the Susceptibility and Progression of COPD in a Spanish Population
Source: PLoS One. 2016 May 10;11(5):e0154998. doi: 10.1371/journal.pone.0154998 (PMC4862690; doi:10.1371/journal.pone.0154998)
Supplement: S5 Table — Data are presented as MAF: minor allele frequency; %: percentage; ORad: adjusted odds ratio; CI: confidence interval; NA: not analyzed. Age, gender and pack-year were included in a multivariate logistic regression analyses as potential independent predictors in an additive model. a rs833069/ rs833070/ rs3025007/ rs3025009/ rs3025010/ rs3025012/ rs3025020/ rs3025032/ rs3025033/ rs3025039/ rs10434. (PDF) [file pone.0154998.s005.pdf]

S5 Table. Replication study of AATATATCACG haplotype in the *VEGFA* gene with COPD susceptibility.

Haplotype[a]	COPD patients vs. Nonsmoking controls			COPD patients vs. Smoking controls		
	**Frecuency (%)**	**OR_{aj} (95%CI)**	**p-value**	**Frecuency (%)**	**OR_{aj} (95%CI)**	**p-value**
GGCGTACTACA	12.26	1	__	16.27	1	__
AATATATCACG	2.55	2.37 (0.40 – 14.03)	>0.05	0.00	0.00 (NA - NA)	1

Data are presented as MAF: minor allele frequency; %: percentage; OR_{ad}: adjusted odds ratio; CI: confidence interval; NA: not analyzed. Age, gender and pack-year were included in a multivariate logistic regression analyses as potential independent predictors in an additive model. [a] rs833069/ rs833070/ rs3025007/ rs3025009/ rs3025010/ rs3025012/ rs3025020/ rs3025032/ rs3025033/ rs3025039/ rs10434.
